# The new clinical standard of integrated quadruple stress echocardiography with ABCD protocol

**DOI:** 10.1186/s12947-018-0141-z

**Published:** 2018-10-02

**Authors:** Eugenio Picano, Quirino Ciampi, Karina Wierzbowska-Drabik, Mădălina-Loredana Urluescu, Doralisa Morrone, Clara Carpeggiani

**Affiliations:** 1 Institute of Clinical Physiology, National Council Research, Via Giuseppe Moruzzi 1, 56124 Pisa, Italy; 2 Fatebenefratelli Hospital of Benevento, Viale Principe di Napoli, 12, 82100 Benevento, Italy; 3 Department of Cardiology, Medical University of Lodz, Bieganski Hospital, Ul Kniaziewicza 1/5, 91-347 Lodz, Poland; 4 CVASIC Research Center Sibiu, “Lucian Blaga” University of Sibiu, Sibiu, Romania; 5 Cardiothoracic department, Cisanello Hospital, University of Pisa, Pisa, Italy

**Keywords:** B-lines, Coronary flow reserve, Echocardiography, Force, Left ventricular contractility, Lung water, Stress echocardiography, Wall motion abnormalities

## Abstract

**Background:**

The detection of regional wall motion abnormalities is the cornerstone of stress echocardiography. Today, stress echo shows increasing trends of utilization due to growing concerns for radiation risk, higher cost and stronger environmental impact of competing techniques. However, it has also limitations: underused ability to identify factors of clinical vulnerability outside coronary artery stenosis; operator-dependence; low positivity rate in contemporary populations; intermediate risk associated with a negative test; limited value of wall motion beyond coronary artery disease. Nevertheless, stress echo has potential to adapt to a changing environment and overcome its current limitations.

**Integrated-quadruple stress-echo:**

Four parameters now converge conceptually, logistically, and methodologically in the Integrated Quadruple (IQ)-stress echo. They are: 1- regional wall motion abnormalities; 2-B-lines measured by lung ultrasound; 3-left ventricular contractile reserve assessed as the stress/rest ratio of force (systolic arterial pressure by cuff sphygmomanometer/end-systolic volume from 2D); 4- coronary flow velocity reserve on left anterior descending coronary artery (with color-Doppler guided pulsed wave Doppler). IQ-Stress echo allows a synoptic functional assessment of epicardial coronary artery stenosis (wall motion), lung water (B-lines), myocardial function (left ventricular contractile reserve) and coronary small vessels (coronary flow velocity reserve in mid or distal left anterior descending artery). In “ABCD” protocol, A stands for Asynergy (ischemic vs non-ischemic heart); B for B-lines (wet vs dry lung); C for Contractile reserve (weak vs strong heart); D for Doppler flowmetry (warm vs cold heart, since the hyperemic blood flow increases the local temperature of the myocardium). From the technical (acquisition/analysis) viewpoint and required training, B-lines are the kindergarten, left ventricular contractile reserve the primary (for acquisition) and secondary (for analysis) school, wall motion the university, and coronary flow velocity reserve the PhD program of stress echo.

**Conclusion:**

Stress echo is changing. As an old landline telephone with only one function, yesterday stress echo used one sign (regional wall motion abnormalities) for one patient with coronary artery disease. As a versatile smart-phone with multiple applications, stress echo today uses many signs for different pathophysiological and clinical targets. Large scale effectiveness studies are now in progress in the Stress Echo2020 project with the omnivorous “ABCD” protocol.

## Background

### Utilization trends of stress echo

**Fig. 1:**
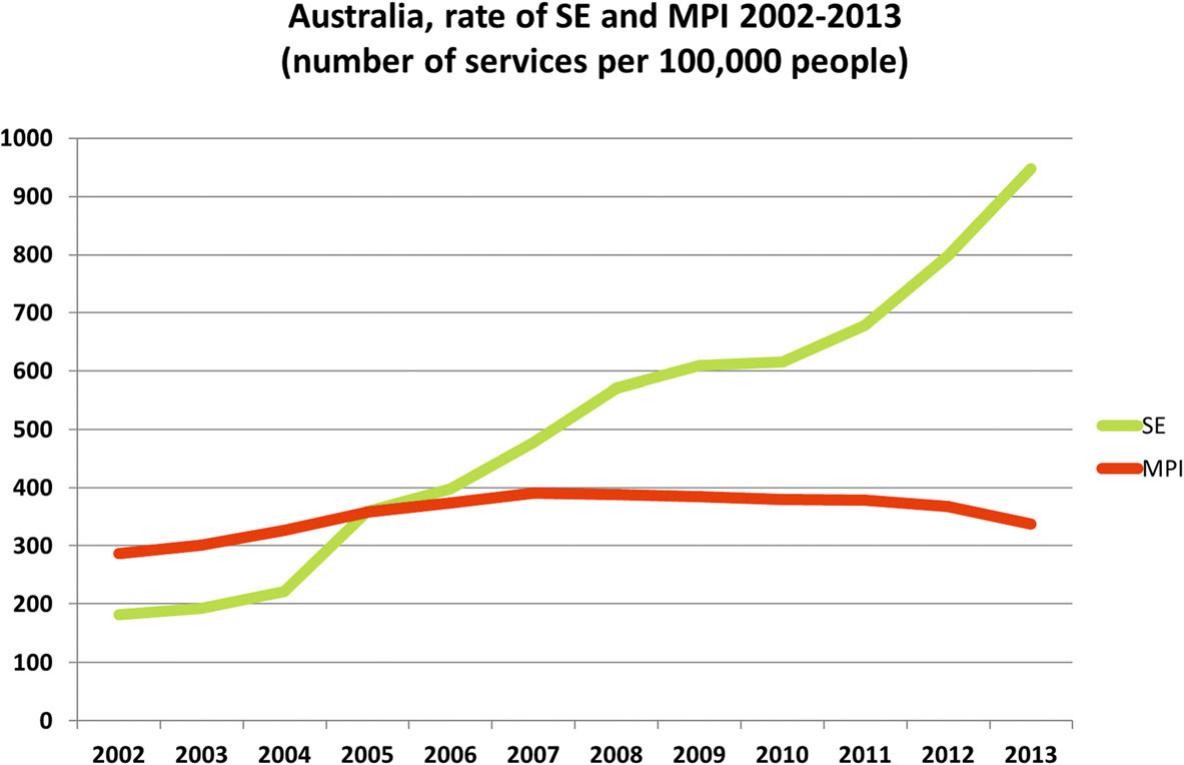
Utilization trends of stress echo. The utilization trends of stress echo compared to myocardial stress scintigraphy in Australia, years 2002–2012 (redrawn from Fonseca et al., ref. [10])

Stress echocardiography (SE) is since decades an established technique for coronary artery disease (CAD) detection and risk stratification [1], with a recognized position in expert recommendations of scientific societies [2, 3] and general cardiology guidelines [4, 5]. In recent years, the new cost-conscious and radiation-conscious climate was the main driver of the observed reduction in myocardial stress scintigraphy and simultaneous growth of SE [6]. In Australia, from 2002 to 2013 the rate of SE use increased 4-fold [7]. In the same time period, the use of other established stress techniques such as stress myocardial scintigraphy remained stable or declined (Fig. 1). In privately insured US patients younger than 65 years, the use of SE increased by 27% from 2005 to 2012 [8]. In Mayo Clinic, the use of myocardial scintigraphy showed a 20-fold rise from 1990 to 1999, but since 2000 SE was introduced and in 2012 the relative utilization rate was 5 SE to 1 scintigraphy [9]. In Ontario, Canada, from 2011 to 2014 the utilization rate of stress scintigraphy decreased at a mean annual rate of - 1.3%, whereas SE in the same period increased at a mean annual rate of + 65.8% [10].

The increased use of SE brings with it an increased risk of inappropriateness [11]. Inappropriate testing is a waste of public health money and also lowers the diagnostic and prognostic value of SE [11–13]. Three indications, account for 79% of all inappropriate testing: symptomatic patients with low pre-test probability of CAD having an interpretable ECG and ability to exercise, asymptomatic patients who had undergone angioplasty less than 2 years before, and asymptomatic patients with low risk [11–14].

### SE and the road to sustainability

The prescription of a single individual cardiac stress imaging test has a large economic, environmental and public health impact since around 10 million stress cardiac imaging test are performed each year in USA only. Small economic wastes, environmental footprint, individual risks of the single inappropriate test multiplied by millions per year represent an avoidable burden for the society and the planet, and a significant population risk [14].

The direct cost of a stress myocardial scintigraphy is 2- to 4- fold higher than SE (5). The difference further widens if we include - as we should - indirect costs due to environmental impact on planet and long-tem costs due to cancer [15].

The environmental impact of a single cardiovascular magnetic resonance or myocardial perfusion scintigraphy is 5- to 100-times higher than that of a SE on human health, ecosystem effects and resource use. One ton of CO_2_ emissions costs 50 US dollars in indirect costs, including contribution to climate change and ozone layer destruction. One echocardiogram produces about 2 kg of CO_2_ and a 3 Tesla magnetic resonance imaging produces 200 to 300 kg of CO_2_ [16, 17]_._

The radiation dose of a single myocardial scintigraphy ranges from 200 to 4000 chest x-rays, whereas there is no radiation exposure for SE or magnetic resonance_._ In terms of population burden, the almost 8 million stress myocardial scintigraphy scans per year in the USA translate into a population risk of about 8 thousand new cancers in the lifetime [18], which represent also an extra-cost of around 50,000 US dollars per cancer [17].

It is therefore not only important the value, but also the cost and the risk of what we are doing in the cardiac imaging lab. In the cost-benefit balance, the cost must include the long-term environmental burden, not only the direct cost. In the risk-benefit balance, the risk must include the long-term cancer risk, not only the acute risks of stress or contrast injection [19]. This obvious concept was a game-changer in the last 15 years [14]. Until 2004, almost no cardiologist knew the radiation doses and risks of what he or she was doing to the patient [20]. Today, mainstream cardiology prescribes that good training should create a culture of respect for radiation hazard and a commitment to minimize exposure and maximize protection [21] .

In this changing scenario, SE plays a key role as the most cost-effective gatekeeper to coronary angiography and ischemia-driven revascularization, associated with fewer downstream coronary angiographies and subsequent risk of adverse events, as shown by a recent meta-analysis including 30 randomized trials on 33,356 patients with low risk acute coronary syndromes or suspected CAD [22]. The strategy centered on functional imaging with SE for prognostic stratification minimizes the radiation exposure associated with an anatomy-first approach by CT coronary angiography complemented by functional testing with scintigraphy, which frequently reaches cumulative exposures in the range of 5000 chest X-rays per patient and sometimes per exam as shown in the Radio-EVINCI international trial [23]. Compared in a randomized manner to exercise-ECG in a low-to- moderate risk population with suspected CAD, a strategy based on upfront SE led to a 20% reduction of costs, due to the combined effects of a reduction in downstream testing, emergency visits and duration of hospital stay [24]. Therefore, a systematic use of SE cuts the costs of downstream testing, deflates the volumes of myocardial scintigraphies, reduces the need for noninvasive and invasive coronary angiography, and puts a substantial barrier, also in medico-legal terms, to the shortcut to anatomy-driven, prognostically futile and inappropriate coronary revascularizations [25]. The optimized and versatile use of SE is an effective way for primary prevention of cancer through the reduction of inappropriate and unjustified use of ionizing testing and therapies [26]. After 25 years of follow-up, 26% of those with positive SE (compared to 17% of patients with negative SE) will die of cancer, and this happens more frequently in patients exposed to higher levels of ionizing testing and therapies [27].We should always minimize the avoidable long-term damage of tomorrow when treating the cardiac patient today, exactly as the oncologists should minimize the prognosis-limiting future cardiac damage when treating cancer with radiotherapy and chemotherapy. A comprehensive risk-benefit analysis should include the chances of long-term damage decades down the line in organs other than those targeted by the primary therapeutic effort.

### The limitations of contemporary SE

In spite of its unsurpassed strengths which make it a dominant technique due to low cost, absence of radiation, environmental friendly nature and versatility coupled with universal availability, SE has important weaknesses that restrict its use and value.

#### The stenotico-centric approach to CAD diagnosis

The vulnerabilities of the patient with CAD and/or heart failure (HF) are multiple and complex, and RWMA is not helpful to capture factors independent from coronary artery stenosis. For any given coronary stenosis or even in the absence of it, the clinical vulnerability to adverse events is more likely in presence of coronary microcirculation abnormalities, alveolar-capillary membrane distress determining increased extravascular lung water, and myocardial scar or fibrosis of the left ventricle (LV) limiting the global contractile reserve [28].

#### Subjectivity of reading

The operator-dependence can be minimized - not abolished - by expert training and adoption of conservative reading criteria, with credentialing via standardized web-based training and certification [29]. Reading harmonization is made easier in the era of connectivity and a second-opinion obtained in real time by senior readers via web and smart-phones [30] can substantially improve the standards of the laboratory, whereas the clinical help of quantitative advanced technologies remains unsettled during stress [2, 3].

#### The low positivity rate of RWMA

The changing profile of patients referred for CAD has dramatically reduced the rate of positive tests based on RWMA, dropped from 70% in the early eighties to < 10% in the first decade of the new millennium [31]. This is due to the higher percentage of patients referred to SE under anti-ischemic therapy, with atypical or absent symptoms and low pre-test probability of disease [31]. The predictive value of the test depends upon the prevalence of the disease in the population under study. The application of SE to a population with 10% prevalence of disease implies that a positive test is associated with a probability of < 50% of having the disease (the so-called “false positive paradox”).

#### The intermediate risk associated with a negative test for RWMA

The risk associated with a negative test is intermediate, not low, and significantly higher than that associated with the negativity of a myocardial perfusion stress test. A recent meta-analysis on 36 studies with 14, 506 patients with known or suspected CAD showed that the hard-event rate (cardiac death and myocardial infarction) in patients with a negative SE for RWMA is still 1.77% per year, which is not so low [32].

#### The limited value of RWMA outside CAD

The population of patients arriving to the SE lab is changing, with a greater percentage of patients with HF, valvular heart disease, adult congenital heart disease, pulmonary hypertension, extreme physiology [33]. In these patients RWMA have little to offer, and the versatility of the technique is largely underused in patients who would certainly benefit from a more comprehensive approach.

### Quadruple imaging IQ-SE: the ABCD protocol

**Fig. 2:**
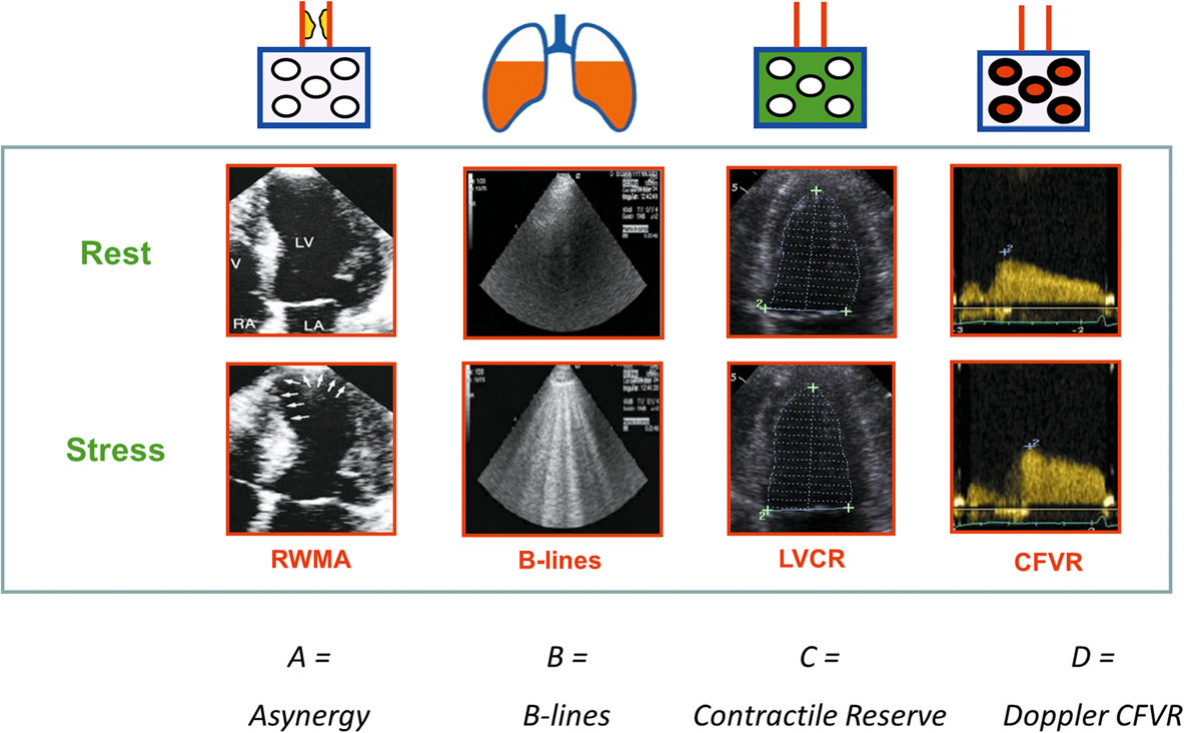
The targets of integrated quadruple imaging stress echo. The 4 patho-physiological targets of IQ-SE: epicardial coronary artery stenosis (with RWMA); lung water (with B-lines); myocardial function (with LVCR); small vessels (with CFVR)

In order to overcome the main limitations of SE based only on RWMA, a new standard of practice has been proposed merging four different parameters with different pathophysiological targets (Fig. 2). In the ABCD protocol, A stands for asynergy and targets a critical epicardial artery stenosis through RWMA; B for B-lines and evaluates pulmonary interstitial edema; C for left ventricular contractile reserve (LVCR) and assesses global myocardial function; and D for Doppler which offers insight into coronary microcirculatory function with coronary flow velocity reserve (CFVR). The main conceptual, methodological and clinical features of the 4 key parameters are shown in Tables 1 and 2.

**Table 1:** The pathophysiological and methodological basis of the IQ-SE protocol

	RWMA	B-lines	LVCR	CFVR
ABCD protocol	A, Asynergy	B, B-lines	C, Contractility	D, Doppler
Target	Epicardial Coronary Stenosis	Lung	Myocardium	LAD stenosis and small vessels
Variable	Ischemia	Water	Force	Flow reserve
Echocardiography	2D	LUS	2-D	PWD
Best projection	4-,3–2 ch	4-site scan	4- and 2-ch	Modified 3-ch
Imaging time	Minutes	Seconds	Seconds	Minutes
Analysis time	Seconds	Seconds	Seconds	Seconds
Feasibility	> 90%	Near 100%	> 95%	> 80%
Evidence	Excellent	Initial	Moderate	Good
Reading	Qualitative	Semi-quantitative	Quantitative	Quantitative
Key parameter	WMSI	B-lines score	ESV	Peak velocity
Abnormal Cut-off	> 1.0	≥ 2.0	< 2.0^a^	< 2.0

^a^< 1.1 in vasodilator stress

**Table 2:** The prognostic potential of the IQ-SE protocol

	Very Low risk	Very High risk
RWMA	Absent	Present
LUS	A-lines	B-lines
LVCR	Preserved	Reduced
CFVR-LAD	Preserved	Reduced
Risk for major events	< 0.5% per year	> 10% per year

The ABCD parameters are conceptually merged, temporally synchronized, and methodologically harmonized in the new standard adopted in SE 2020 study: the Integrated Quadruple (IQ)-SE [34].

The technical challenges are not much greater than that posed by imaging and assessment of RWMA alone. Some of the new parameters are faster to image and simpler to measure than the old ones. The acquisition of diagnostic images is the simplest for B-lines, simple with LVCR, not-so-simple for RWMA and more difficult for CFVR. The image analysis is the simplest for B-lines and CFVR, not-so-simple for LVCR and more difficult for RWMA. All in all, integrating the required training for image acquisition and analysis, B-lines can be considered the kindergarten, LVCR the primary (for acquisition) or secondary (for analysis) school, RWMA the university, and CFVR the PhD course of the SE *cursus studiorum*.

### A for regional wall motion asynergy: ischemic or non-ischemic heart

#### The conceptual meaning

**Fig. 3:**
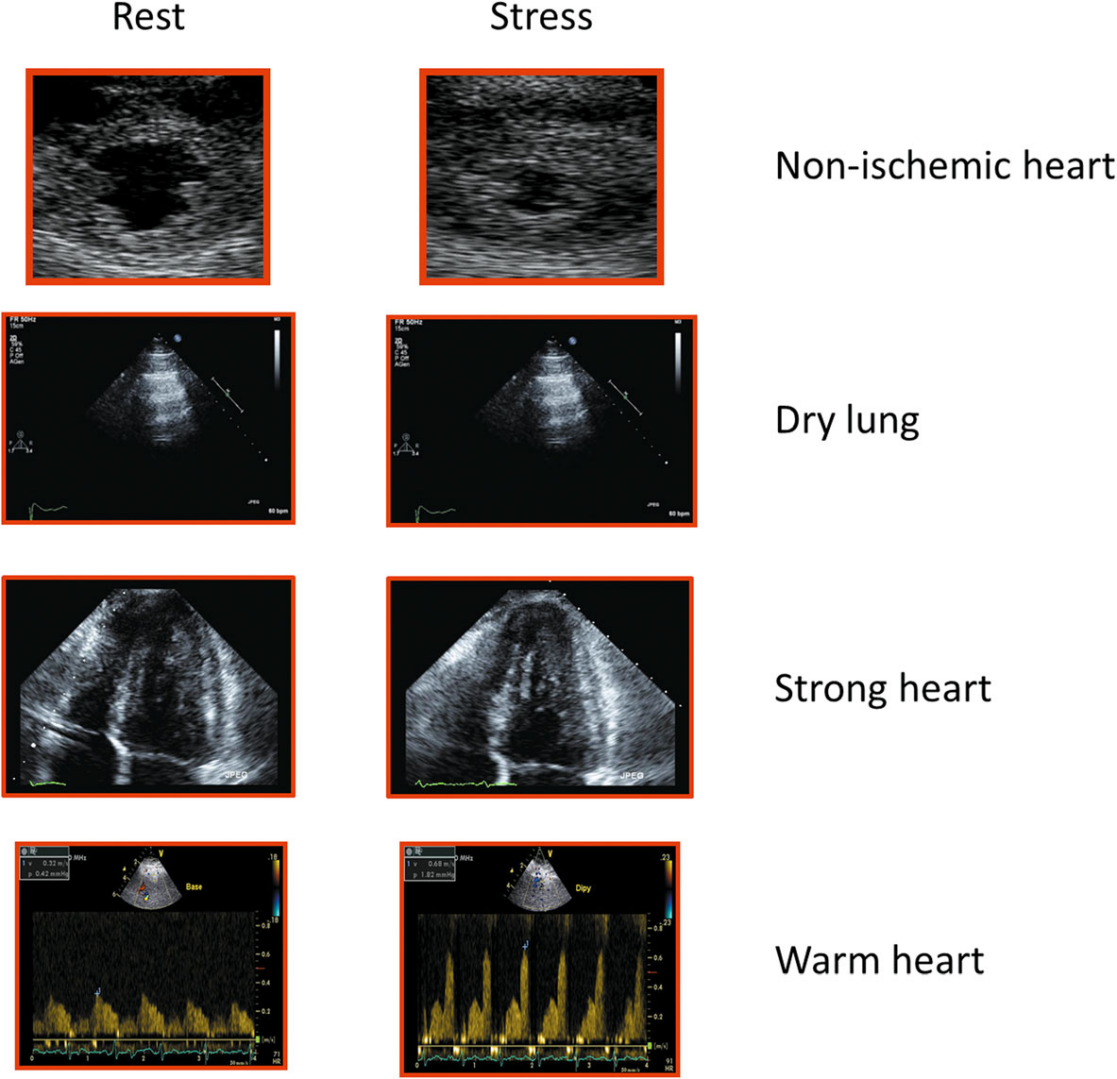
The normal quadruple imaging response. The normal IQ-SE response of a non-ischemic (*first row*), dry (*second row*), strong (*third row*) and warm (*fourth row*) heart

**Fig. 4:**
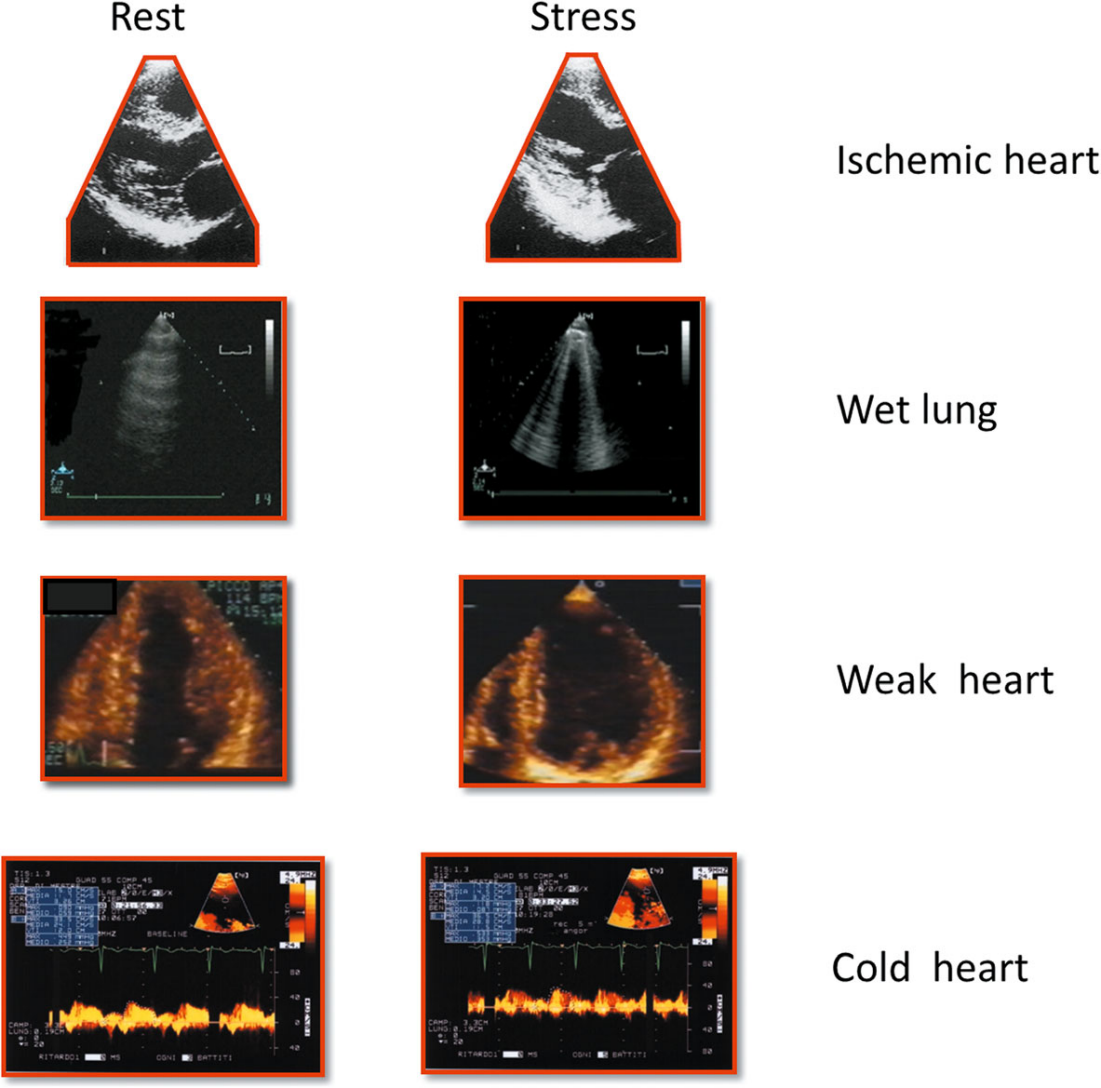
The abnormal quadruple imaging response. The abnormal IQ-SE response of an ischemic (*first row*), wet (*second row*), weak (*third row*) and cold (*fourth row*) heart

The cardinal sign of transient myocardial ischemia is a stress-induced regional asynergy (also called dyssynergy) in its three degrees: hypokinesia (reduction of systolic motion and thickening); akinesia (absence of systolic thickening and motion); and dyskinesia (paradoxical systolic movement and systolic thinning) [1]. The absence of RWMA identifies a non-ischemic heart (Fig. 3, first row); its presence an ischemic heart (Fig. 4, first row). Ischemia is required for RWMA, but even under ideal imaging conditions RWMA can occur without ischemia (for instance in left bundle branch block or pacemaker stimulation from the right ventricle or myocardial fibrosis in non-ischemic dilated cardiomyopathy), and ischemia (or even infarction) can occur without RWMA. In fact, the detection of RWMA requires a critical ischemic mass of at least 20% of transmural wall thickness and about 5% of the total myocardial mass. Therefore, relatively milder and more localized forms of ischemia do not leave echocardiographic fingerprints on RWMA [1] .

#### Pathophysiology

RWMA are linked to stress-induced subendocardial hypoperfusion, usually caused by a critical reduction in coronary flow reserve due to an anatomically and physiologically significant epicardial artery stenosis [1]. A reduction in subendocardial blood flow of 20% produces a 20% decrease in regional wall thickening (mild hypokinesis); a 50% reduction in subendocardial blood flow a 40% reduction in wall thickening (severe hypokinesis); and when subendocardial blood flow is reduced by 80%, akinesia occurs. When the flow reduction is extended to the subepicardial layer, dyskinesia occurs [1].

In viable segments with resting RWMA, stress will normalize function through an increase in flow. This allows to separate viable from necrotic segment, the latter showing no improvement after stress with a fixed wall motion response (akinesis at rest- unchanged after stress). The possibility of recruiting the inotropic reserve might appear paradoxical in the presence of hibernation or stunning. The traditional concept is that a decrease in resting coronary blood flow indicates that coronary vasodilating reserve is exhausted. However, hibernating and stunned segments have some residual coronary vasodilatory reserve, which is mirrored by contractile reserve. The physiology of myocardium is that of an erectile organ, and in the low flow range the increase in flow is paralleled by an increase in regional function [35].

#### Methodology

RWMA is summarized in the Wall Motion Scored Index, with a segmental score from 1 = normal/ hyperkinetic to 4 = dyskinetic in a 16- or 17-segment model of the LV (2, 3). Stress values of peak Wall Motion Score Index may range from normal (rest = stress = 1.0) to mild (1.07–1.40), moderate (1.41–1.69), and severe (≥1.70) left ventricular dysfunction, with higher values associated with worse outcome [36].

#### Clinical evidences

RWMA are more specific (around 90%) but less sensitive (around 80%) than perfusion abnormalities for the detection of CAD, and this is true regardless of the employed stress or imaging technique [2–5]. The ischemic response by RWMA is a strong prognostic predictor of subsequent hard events and death in all patients subsets, from low risk patients with stable angina and preserved baseline left ventricular function to patients with known CAD and previous myocardial infarction [2–5]. The risk stratification can be improved with the combination of RWMA with clinical parameters, if we consider 6 simple items to build a score ranging from zero to 6 which acts as a multiplier of SE risk. The considered items are: age > 65 years; male sex; diabetes; left bundle branch block; anti-anginal therapy at the time of testing; wall motion abnormalities at rest [37]. The hard-event rate of a negative SE increases 20-fold in a negative SE and 100-fold in a positive SE when going from 0 to 1 to 5–6 clinical risk factors.

In patients with reduced resting left ventricular function, an improvement in regional wall motion during SE (“viability response”) is associated with a better survival in different groups of patients: early after acute transmural myocardial infarction, chronic ischemic CAD, non-ischemic dilated cardiomyopathy on medical therapy [2–5] or treated with cardiac resynchronization therapy [38].

### B for B-lines: wet or dry lung

#### The conceptual meaning

The normal A-profile (normal lung sliding with A-lines) detected with lung ultrasound (LUS) identifies a relatively dry lung (Fig. 3, second row) with < 500 mL of extravascular lung water. The B-profile (normal lung sliding and B-lines increasing during stress) a wet lung (Fig. 4, second row) with abnormal accumulation of extravascular lung water [39].

#### Pathophysiology

The B-profile with normal lung sliding and B-lines provides a unique way to evaluate semi-quantitatively subclinical pulmonary congestion which heralds impending acute HF and cannot be assessed reliably with standard approaches of measuring weight gain, pulmonary crackles on lung auscultation, or Kerley B-lines on chest X-rays [40].

#### Methodology

LUS focused on B-lines usually starts within 5 s of the end of exercise, or antidote administration in pharmacological stress. The 4-site simplified scan requires on average 20 s, and is equally accurate than the more time-consuming 28-site scan previously adopted [41]. Scanning is performed on the anterior and lateral hemithoraces, from midaxillary to mid-clavicular lines on the third intercostal space. Each site is scored from 0 (A-lines) to 10 (white lung of coalescing B-lines). The cumulative score range is from 0 to 40, with delta (∆, stress- rest) values < 2 considered normal. The intra- and inter-observer variability are < 5 and < 10% respectively. An increase of B-lines during stress ≥2 is significant (abnormal). Stress B-lines values may range from normal dry lung (rest = stress = 0–1), or wet lung with mild (2–5), moderate (6–9), and severe (≥10) accumulation of extravascular lung water.

#### Clinical evidences

B-lines during stress are detectable in the majority of patients with HF and either reduced [42] and preserved ejection fraction (EF) [43], and may also appear in CAD patients during physical or pharmacological stress [44]. Their appearance or worsening during stress is associated with higher resting levels of cardiac natriuretic peptides, lower anaerobic threshold during spiroergometry testing, and a worse outcome, with higher mortality and rate of re-hospitalization for acute decompensated HF [44]. In patients with CAD, stress B-lines are associated with extensive RWMA but may occur also in patients with normal left ventricular function and normal coronary arteries, in presence of severe mitral regurgitation, systolic blood pressure > 200 mmHg or increased systolic pulmonary artery pressure due to diastolic dysfunction [44]. Rest and especially (further upstream) stress B-lines are an early event in the pre-symptomatic “lung water cascade” of events eventually leading from increase in left ventricular filling pressures to pulmonary congestion and clinical decompensation. They might eventually become a specific marker of cardiac origin of dyspnea in the same way as a RWMA is today considered a highly specific marker of ischemic origin of chest pain [45].

A significant number of patients can show B-lines at rest and during stress due to interstitial lung disease. However, these fibrotic (“dry”) B- lines do not change with exercise, differently from watery (“wet”) B-lines which increase with exercise and decrease with diuretics [44].

### C for left ventricular contractile reserve: weak or strong heart

#### The conceptual meaning

The left ventricular force (also called elastance) is a measure of the intrinsic contractile state of the ventricle [46]. The presence of preserved LVCR during SE identifies a strong heart with higher values of peak stress force and smaller LV end-systolic volume (ESV) than baseline (Fig. 3, third row). An abnormal LVCR is associated with a weak heart with lower peak values of force and larger LV ESV at peak stress than baseline (Fig. 4, third row). LVCR contains information on left ventricular volumes and systolic blood pressure missed by RWMA, and on the other side RWMA gives an information on subendocardial layer perfusion missed by LVCR, which is usually normal in presence of localized hypoperfusion, also for the compensatory hyperfunction of non-ischemic regions or layers. The heart can be ischemic but strong, and non-ischemic yet weak.

#### Pathophysiology

Differently from ejection fraction (EF), LVCR is not (or less) affected by changes in preload, afterload and heart rate. The conventional definition of contractile reserve by ≥5 points increase in EF only in 40% of cases agrees with LVCR defined by force [47]. Force definition incorporates two well recognized prognostic markers, since low systolic blood pressure response and increased LV ESV during stress [48] both determine a blunted force response and have been separately associated with increased mortality.

#### Methodology

The force is measured as the ratio of end-systolic pressure (by cuff sphygmomanometer)/ESV (by 2D echocardiography). The calculation of ESV by 2D echocardiography is a relatively precise measurement, with > 90% measurements within 10% difference, and a substantially lower inter-observer variability of ESV than end-diastolic volume [49, 50]. LVCR is the peak stress/rest ratio of left ventricular force. LVCR values during dobutamine or exercise stress may range from normal (> 2.0) to mild (1.5–2.0), moderate (1.01–1.49), and severe (≤1.0) dysfunction. Values are shifted towards lower values (abnormal < 1.1) for vasodilator stresses [47].

#### Clinical evidences

LVCR is highly feasible during all forms of stress: exercise [51], pacing [52], dobutamine [53] and dipyridamole [54]. In patients with stable angina and normal resting left ventricular function, LVCR reduction during dipyridamole stress showed a 86% sensitivity and 87% specificity for the detection of angiographically assessed CAD [55]. When outcome is the gold standard, LVCR reduction outperforms RWMA, ∆-WMSI and ∆-EF in predicting adverse events including death [53, 56, 57]. In absence of RWMA, an impaired LVCR is more often present with underlying critical CAD and/ or myocardial scar in brain-dead marginal heart donors who underwent autoptic verification after stress [54].

### D for Doppler flowmetry in coronary flow velocity reserve: warm or cold heart

#### The conceptual meaning

The increase in coronary blood flow during hyperemia can be conceptually associated with changes in the local myocardial temperature. Experimental and clinical studies show that a decrease in blood flow leads to a drop of regional myocardial temperature, and an increase leads to the immediate appearance of warm spots detectable by a noninvasive thermogram [58]. Therefore the presence of a preserved CFVR identifies a warm heart (Fig. 3, fourth row), whereas an abnormal (reduced) CFVR is associated with a cold heart (Fig. 4, fourth row).

#### Pathophysiology

CFVR can be impaired in presence of a physiologically significant epicardial artery stenosis and in this case is usually - but not always - accompanied by RWMA [33]. A blunted CFVR often occurs with normal coronary arteries in absence of RWMA, in presence of an altered coronary microcirculation, as it can be found for instance in non-ischemic dilated cardiomyopathy, hypertrophic cardiomyopathy, HF with normal EF, aortic stenosis, acute rejection of transplanted heart and several other conditions [3, 33].

#### Methodology

Noninvasively assessed CFVR obtained from TTE is tightly correlated with invasively assessed coronary flow reserve [59]. Acquisition of pulsed-wave Doppler of coronary flow velocity on LAD is performed at baseline and peak stress, usually just before RWMA and LVCR. It is the least feasible and the more technically demanding of the new parameters: it can be obtained in over 90% of patients on mid-distal LAD, but requires state-of-the art technology and dedicated training. Once properly acquired, it is easy to measure and has simple, stress-independent prognostic cut-offs (abnormal values < 2.0). Although most data have been obtained with vasodilator stress [59], which is by far technically easier and therefore more popular for assessing CFVR, similar data can be obtained with dobutamine [60] and with semi-supine exercise stress [61] . Stress values may range from normal (> 2.0) to mild (1.7–2.0), moderate (1.41–1.69), and severe (≤1.40) dysfunction [59].

#### Clinical evidences

In patients with suspected CAD, CFVR has high sensitivity but limited specificity for CAD detection and only targets the left anterior descending coronary artery, which limits the diagnostic usefulness [59] . The main interest in CFVR is therefore for risk stratification. Patients with negative SE identified on the basis of absence of RWMA can be reclassified as at intermediate risk if a reduced CFVR is present. A reduced CFVR predicts a higher mortality, is not affected - differently from RWMA [62] - by concomitant anti-anginal therapy [63] and has additional and independent prognostic value over RWMA in different patients’ subsets, including stable coronary artery disease [63], non-ischemic dilated cardiomyopathy [64], hypertrophic cardiomyopathy [65], and asymptomatic moderate-to-severe aortic stenosis with normal EF and normal coronary arteries [66]. The prognostic information is incremental over that provided by myocardial perfusion scintigraphy in patients with suspected CAD [67].

### Risk stratification of SE results beyond regional wall motion abnormalities

**Fig. 5:**
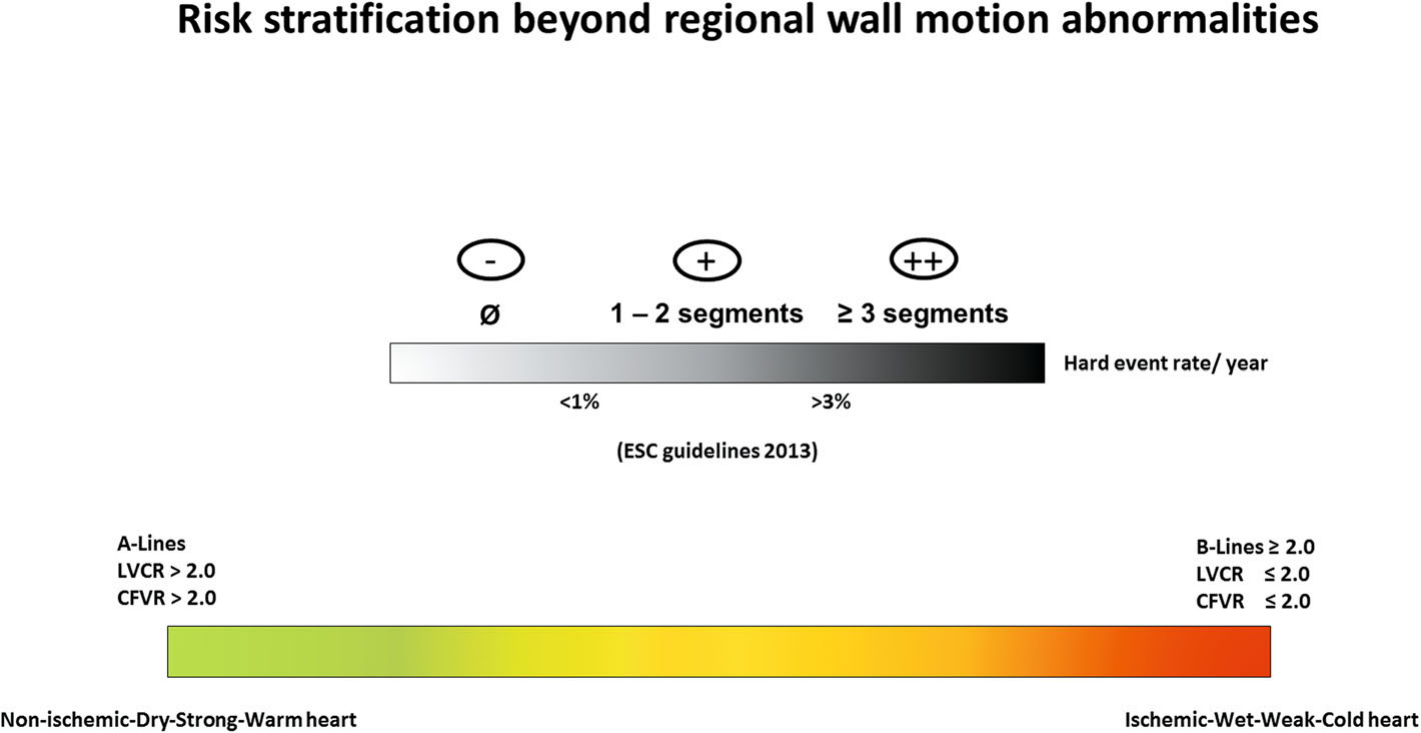
The risk stratification with quadruple imaging. The risk stratification with SE, from binary (black or white) response based only to RWMA endorsed by current guidelines (*upper row*) to the spectrum of responses (from green of lowest to red of highest risk) obtained by quadruple imaging with RWMA supplemented with B-lines, LVCR and CFVR

The integration of 4 different variables into a single one-stop shop expands the risk stratification potential of SE. The current approach to risk stratification is based on presence or absence of RWMA (Fig. 5, upper row). This approach is the only possible evidence-based strategy today [4, 5] but clearly under-uses the unique versatility of SE when dual and triple imaging are applied. In 103 patients with HF and reduced EF studied with dual (RWMA+B-lines) imaging, during a median follow-up of 8 months new major events occurred in 36% of patients without exercise-induced RWMA with moderate-to-severe B-lines, and only in 5% of those with absent or mild stress B-lines [42]. In 91 patients with HF due to idiopathic dilated cardiomyopathy studied with dual (RWMA+LVCR) imaging, event rate at 18 months was 4% in patients with, and 37% in patients without preserved LVCR [53]. In 4313 patients with known or suspected stable CAD studied with dual (RWMA+ CFVR) imaging, the 4-year mortality associated with a negative test for RWMA was 3% in patients with preserved CFVR and 12% in those with reduced CFVR [68]. In 375 diabetic patients without dipyridamole-induced RWMA and studied with triple imaging (RWMA+ LVCR+CFVR), the rate of hard events at 3-year follow-up was 3% in patients with both normal CFVR and LVCR, 5-fold higher in patients with abnormality of either CFVR or LVCR, and 9-fold higher in patients with both abnormal CFVR and LVCR [69]. The black and white risk stratification becomes color-coded with a spectrum of responses (from benign all-negative green-code to malignant all-positive red-code) (Fig. 5, lower row).

### The ABCD protocol in diastolic stress echocardiography

A specific and challenging aspect for SE is the diagnosis of diastolic dysfunction. Diastolic SE is useful in patients with unexplained shortness of breath, exertional fatigue or poor exercise capacity, with normal left ventricular ejection fraction, high cardiac natriuretic peptides, especially in presence of cardiovascular risk factors (advanced age, arterial systemic hypertension, diabetes mellitus, obesity, sedentary lifestyle) and structural alterations of resting TTE such as left atrial volume index dilation and left ventricular hypertrophy. SE is currently recommended in patients falling in the normal or gray zone of diastolic function at rest, as determined by an integration of several parameters: mitral velocities (E, E wave deceleration time, A and E/A ratio), mitral annular velocity (e’), E/e’ ratio, peak velocity of tricuspid regurgitant jet and left atrial volume index [70]. During exercise, tricuspid regurgitant jet velocity and E/e’ are considered the most valuable parameters, and their increase suggests the presence of pulmonary hemodynamic congestion and increased LV filling pressures indicative of diastolic dysfunction during stress [71] .

Recent data suggest that the current approach based mainly on tricuspid regurgitant jet velocity and E/e’ can suffer from substantial feasibility and accuracy problems [50, 72]. Other parameters may be more feasible and possibly more useful during stress, such as acceleration time of pulmonary flow and B-lines. Acceleration time is measured as the time interval between onset of systolic flow to peak flow, and the normal value is > 105 ms [73]. It decreases with increases in pulmonary artery mean and systolic pressures [74]. The initial clinical experience demonstrates a substantially higher feasibility of acceleration time compared to tricuspid regurgitant jet velocity during stress [75]. Experimental data suggest that the presence and progression of HF with preserved EF is accompanied by shortening of acceleration time and increase of B-lines [76].

Therefore, the ABCD protocol has potential to be applied also in diastolic SE, since the regional wall motion abnormalities (A) must be ruled out in the initial evaluation with SE to screen the origin of dyspnea due to ischemia or mitral regurgitation or left ventricular outflow tract obstruction. B-lines can be present as a hallmark of the cardiogenic origin of dyspnea and have a specific, attractive potential in this specific application [43]. Left ventricular contractile reserve (C) can be useful to detect a subclinical occult systolic dysfunction in a subset of these patients [77]. D might be helpful for a more comprehensive pathophysiological and prognostic assessment of these patients, who are known to have a reduced coronary flow reserve associated with a worse outcome [78]. In addition to the core ABCD protocol, new parameters can be added. E for E/e’ and end-diastolic volume to identify a limited diastolic volume reserve and reduced LV compliance during stress, with higher E/e’ values for lower end-diastolic volumes compared to normals [79]. F for tricuspid regurgitant or pulmonary systolic forward Flow, possibly complementing each other for assessing pulmonary hypertension. The standard ABCD protocol may become ABCDEF protocol for diastolic SE, but prospective validation of this working hypothesis is needed at this point.

## Conclusion

With the ABCD protocol, IQ-SE separates ischemic hearts with RWMA from non-ischemic hearts without RWMA; dry lungs with A-lines from wet lungs with B-profile; strong hearts with normal LVCR and reduced ESV from weak hearts with blunted LVCR and dilated ESV; and warm hearts with preserved CFVR from cold hearts with reduced CFVR. The new BCD parameters need minimal extra-imaging and extra-analysis time, but the potential benefits are extraordinary, since IQ-SE gains versatility and objectivity, increases the positivity rate, expands the domain of application of SE from CAD to HF patients, and improves the risk stratification potential. The annual hard-event rate of a test with quadruple negativity (non-ischemic, dry, strong and warm heart) is substantially lower than that associated with a test with quadruple positivity (ischemic, wet, weak and cold heart). All possible combinations of intermediate responses can be found in between the highest risk (quadruple positivity) and lowest risk (quadruple negativity) pattern [34].

In addition to the universal IQ-SE protocol, other parameters can be added in special subsets. As it is not possible to assess all variables during stress in all patients, the parameters of potential interest should be prioritized for the individual patient on the basis of the perceived importance of each [33]. Priority will be given to right ventricular function in patients with repaired tetralogy of Fallot, to pulmonary hemodynamics in patients with primary or secondary pulmonary hypertension, to valve gradients and regurgitation in patients with valvular heart disease [80], and to intraventricular gradients in patients with HCM [33, 71].

The old landline telephone with a single sign (RWMA) for one patient with known or suspected CAD [81] is now a versatile smart-phone with multiple applications, and can be tailored in the individual patient according to clinical needs. Large scale effectiveness studies with IQ-SE are now under way with the Stress Echo 2020 project [82], and will hopefully provide the evidence needed for large scale acceptance of the omnivorous (with all variables) and ubiquitous (for all patients) “ABCD” protocol.

## Abbreviations

CAD: Coronary artery disease
CFVR: Coronary flow velocity reserve
EF: Ejection fraction
ESV: End-systolic volume
HF: Heart failure
IQ-SE: Integrated quadruple stress echocardiography
LUS: Lung ultrasound
LV: Left ventricle
LVCR: Left ventricular contractile reserve
RWMA: Regional wall motion abnormalities
SE: Stress echocardiography
